# *Drosophila* enabled promotes synapse morphogenesis and regulates active zone form and function

**DOI:** 10.1186/s13064-020-00141-x

**Published:** 2020-03-17

**Authors:** Elizabeth M. McNeill, Cheryl Thompson, Brett Berke, Vivian T. Chou, Jannette Rusch, April Duckworth, Jamin DeProto, Alicia Taylor, Julie Gates, Frank Gertler, Haig Keshishian, David Van Vactor

**Affiliations:** 1 Department of Food Science and Human Nutrition, Iowa State University, Ames, IA USA; 2 Department of Cell Biology and Program in Neuroscience, Blavatnik Institute, Harvard Medical School, Boston, MA USA; 3 Department of Biology, Yale University, New Haven, CT USA; 4 Department of Biology, Bucknell University, Lewisburg, PA USA; 5 Department of Biology, Massachusetts Institute of Technology, Cambridge, MA England

**Keywords:** *Drosophila*, Actin, Synapse, Ena/VASP, Dlar, Receptor protein tyrosine phosphatase

## Abstract

**Background:**

Recent studies of synapse form and function highlight the importance of the actin cytoskeleton in regulating multiple aspects of morphogenesis, neurotransmission, and neural plasticity. The conserved actin-associated protein Enabled (Ena) is known to regulate development of the *Drosophila* larval neuromuscular junction through a postsynaptic mechanism. However, the functions and regulation of Ena within the presynaptic terminal has not been determined.

**Methods:**

Here, we use a conditional genetic approach to address a presynaptic role for Ena on presynaptic morphology and ultrastructure, and also examine the pathway in which Ena functions through epistasis experiments.

**Results:**

We find that Ena is required to promote the morphogenesis of presynaptic boutons and branches, in contrast to its inhibitory role in muscle. Moreover, while postsynaptic Ena is regulated by microRNA-mediated mechanisms, presynaptic Ena relays the output of the highly conserved receptor protein tyrosine phosphatase Dlar and associated proteins including the heparan sulfate proteoglycan Syndecan, and the non-receptor Abelson tyrosine kinase to regulate addition of presynaptic varicosities. Interestingly, Ena also influences active zones, where it restricts active zone size, regulates the recruitment of synaptic vesicles, and controls the amplitude and frequency of spontaneous glutamate release.

**Conclusion:**

We thus show that Ena, under control of the Dlar pathway, is required for presynaptic terminal morphogenesis and bouton addition and that Ena has active zone and neurotransmission phenotypes. Notably, in contrast to Dlar, Ena appears to integrate multiple pathways that regulate synapse form and function.

## Background

The synapse is an essential functional unit of all neural circuits. During nervous system development, synaptic architecture is established through a coordinated process of morphogenesis and cell-cell interaction, thus consummating specific connections between pre- and post-synaptic cells [1–3]. In addition to its critical role in animal development, synapse morphogenesis underlies the activity-dependent plasticity and remodeling of neural circuitry. Accordingly, numerous signaling networks control synapse morphogenesis. The actin cytoskeleton is among the major targets of these signaling pathways, and it drives multiple aspects of synapse structure and function [4–6]. While the importance of actin assembly to synaptogenesis is clear, our knowledge of the key effector proteins and upstream signaling pathways is rapidly expanding [4–6].

A key actin regulator that has emerged as a promising link between signaling networks and mechanistic changes in the synaptic cytoskeleton is the actin-regulatory protein Enabled (Ena), a founding member of the highly conserved Ena/VASP (Vasodilator-Stimulated Phosphoprotein) family of proteins [7, 8]. Ena/VASP proteins localize to leading edge membranes and sites of cell-cell or cell-matrix interaction, where they can promote or inhibit membrane protrusion depending on the organization of the microfilament network [7, 8]. The signature domains of Ena include an N-terminal Ena/VASP Homology 1 (EVH1) localization domain, a central proline-rich region motif, and a C-terminal EVH2 actin assembly domain [7, 8]. Through these domains, Ena promotes F-actin barbed end assembly by recruiting actin monomers while suppressing the function of actin capping proteins [7, 8].

In the nervous system, Ena/VASP proteins are best known for their roles in neuronal process formation, growth cone migration, and axonal guidance [9]. Recent studies also highlight important roles at the synapse [10, 11]. Mammalian Ena (Mena) and VASP localize in dendritic spines where they interact with scaffolding molecules in the postsynaptic cytomatrix [12, 13]. A similar postsynaptic co-localization of *Drosophila melanogaster* Ena with Discs-large (Dlg), the fly ortholog of PSD-95, is observed at the glutamatergic neuromuscular junction (NMJ) [14]. In this postsynaptic compartment, conserved Ena/VASP C-terminal domains are required to promote the growth of the postsynaptic membrane folds known as subsynaptic reticulum (SSR), and to restrict the growth of the presynaptic arbor [14, 15]. Precise control of postsynaptic Ena activity in *Drosophila* muscle is mediated by the microRNA miR-8 [14, 15]. In the presynaptic compartment, Ena is required to prevent ectopic formation of satellite boutons, which are abnormal, undersized boutons observed in many NMJ growth mutants, possibly by regulating the balance of linear versus branched actin polymerization and assembly [16]. However, additional roles of presynaptic Ena at the terminal arbors have not been defined.

Clues as to the nature of presynaptic Ena function and regulation in *Drosophila* have come from analyses of its interactions with potential upstream factors. For instance, regulation of satellite boutons by Ena occurs downstream of the Strip-Hippo signaling pathway [16], consistent with Hippo-mediated regulation of Ena in the fly ovary [17]. Ena is also a known downstream target of the Abelson (Abl) non-receptor tyrosine kinase, which in *Drosophila* restricts the growth of presynaptic arbors and regulates neurotransmitter release [18]. Ena was originally identified in a screen for suppression of Abl lethality [19]. Ena binds to the Abl SH3 protein interaction domains through its proline-rich motifs and is a substrate of the Abl catalytic domain [20–23]. In *Drosophila,* Abl antagonizes Ena function during axon guidance [24–27], and Abl and Ena co-expression in *Drosophila* cultured cells redistributes subcellular F-actin puncta, unlike Ena expression alone [18]. Unfortunately, the early lethality of *ena* null mutants [28] precludes examination of Ena’s role in synaptic development, including its potential interaction with Abl.

*Drosophila* Ena is a substrate and intracellular binding partner of the highly conserved LAR (Leukocyte common antigen related) receptor protein tyrosine phosphatase (RPTP) also reported as Dlar [25, 29]. Dlar-family RPTPs are potent modulators of synapse morphogenesis from ecdysozoa to vertebrata [30, 31]. Previous analysis of Dlar demonstrated that catalytic RPTP activity was required in neurons to promote NMJ growth, suggesting that dephosphorylation of Ena might be essential for bouton addition [29]. Because Dlar is antagonistic to Abl for NMJ arbor growth and active zone development [29, 32], we hypothesized that Ena would mediate some aspect of the functions of both Dlar and Abl at the *Drosophila* NMJ. In the current study, we test this hypothesis using conditional loss of function (LOF) methods. Our data indicate that Ena-dependent larval NMJ expansion is epistatic to Dlar and Abl, whereas Abl is epistatic to Dlar. These results suggest that Ena function is critical for presynaptic terminal morphogenesis and bouton addition under control of the Dlar pathway. Separate from its role in synapse morphogenesis, Ena also suppresses neurotransmission such that its loss enhances the amplitude and frequency of spontaneous glutamate release with no effect on evoked release. In contrast to Dlar, Ena appears to integrate multiple pathways that regulate synapse form and function.

## Methods

### Drosophila genetics

All stocks were maintained and crossed at 25 °C according to standard procedures. Stocks were obtained from the Bloomington Stock Center (Bloomington, IN, USA) unless otherwise specified. 1407-Gal4 [33] was used to drive pan-neural expression. Embryos carrying *UAS-mito-FP*_*4*_ and *1407-Gal4* driver as well as the *UAS-mito-AP4* control were hatched at 18 °C, at which Gal4-expression is suppressed, thus preventing embryonic expression of *UAS-mito-FP*_*4*_ and avoiding early embryonic axon guidance phenotypes. Animals were then shifted to 25 °C to promote expression of UAS-constructs starting in the first instar stage. The following lines were previously published: *UAS-mito-FP*_*4*_*-EGFP* and *UAS-mito-AP*_*4*_*-EGFP* [34]; *UAS-Ena(+)* [25]; *enaGC5/+* [23]; *Dlar*^*5.5*^ and *Dlar*^*13.2*^ [35]; *SdcP*, *Df48,ubsara* [36, 37]. Abl lines were a gift from F.M. Hoffman.

### Immunohistochemistry and quantification of NMJ development

Wandering third instars were dissected in cold Ca^2+^-free saline and fixed in cold 4% paraformaldehyde for 20 min. Dissected pelts were washed in PBS + 0.1% Triton-X 100, blocked for 1 h in 5% heat-inactivated goat serum (Millipore, Burlington, MA), incubated overnight at 4 °C in primary antibody diluted in block, washed in PBS + 0.1% Triton-X 100, and incubated for 3 h in secondary antibody diluted in PBS + 0.1% Triton-X 100. All steps were performed at room-temperature unless otherwise noted. The following primary antibodies were obtained from the Developmental Studies Hybridoma Bank Iowa City, IA, USA: anti-Futsch (1:100), and anti-Dlg (1:50). The following primary antibodies were also used: anti-horseradish peroxidase (HRP, 1:1200, Jackson ImmunoResearch, West Grove, PA, USA); endophilin (1:2000, H. Bellen [38]); GluRIII (1:5000, A. DiAntonio [39]). Secondary antibodies conjugated with fluorophores Alexa 488, Alexafluor 568 (1400, Life Technologies, Grand Island, NY, USA) were used at a 1:400 dilution. Motor neuron terminals of muscle 6 and 7 in the abdominal segment A2 of wandering third instar larvae were used for the quantification of all morphological parameters. Both type 1b and 1 s boutons were included in bouton number counts. A branch is defined as any branch of two or more boutons off of the primary nerve terminal and any subsequent branches off of these secondary branches. Gross bouton size appeared unchanged across genotypes. This analysis was carried out using a Zeiss Axioplan2 microscope and a Hamamatsu ORCA wide-field digital camera as previously described in [15].

### Confocal and structured illumination microscopy

Confocal microscopy was performed using a Nikon A1 confocal inverted microscope. Confocal images of synaptic boutons of 6/7 neuromuscular junctions were taken at 40X magnification. Prior to acquisition, laser parameters were adjusted to obtain non-saturating conditions. Structured Illumination microscopy was completed using a Nikon N-SIM Super Resolution Microscope. Images were captured with PCO front illuminated sCMOS camera. Image reconstruction and analysis of Bruchpilot (Brp) staining were completed using Image J software.

### Electron microscopy

Wandering third instar larvae were dissected in Ca2 + −free saline. The gut and internal organs were removed. Larvae were then fixed in 2.5% paraformaldehyde, 5.0% glutaraldehyde, and 0.06% picric acid in 0.1 M cacodylate buffer overnight at 4 °C, and rinsed three times for 20 min on ice in 0.1 M cacodylate buffer. Brain and other debris were removed and the A1-A3 muscle area was cut out for further processing. The samples were then post-fixed with 1% osmium tetroxide and 1.5% potassium ferrocyonide in 0.1 M cacodylate buffer for 1 h on ice. Samples were then rinsed three times for 5 min in deionized water, washed in maleate buffer twice for 10 min, incubated in 1% uranyl acetate in maleate buffer for 1 h, and dehydrated in ethanol series (50, 70, 95, 100 and 100%) for 10 min each. Then samples were rinsed in propylene oxide 20 min twice, and incubated in 1:1 propylene oxide and TAAB resin solution overnight. Finally, they were embedded in fresh resin at 65 °C until hard. Sections were cut parallel to the surface of the muscle. Once an A2 6/7 muscle 1b bouton was identified, 50 nm sections were taken for a total of 5 μm. Sections were mounted on single slot grids, stained with lead and uranyl acetate, and imaged on a JEOL 1200EX – 80 kV electron microscope at 6500× and 25,000× magnification. Fifty nm serial sections cut perpendicular to the surface of the muscle in an anterior to posterior orientation of the larvae were used to obtain mean active zone area.

### Electrophysiology

Third instar larvae were dissected and recorded as previously described [40]. Briefly, the recording saline contained 140 mM NaCl, 5 mM KCl, 1 mM CaCl2, 4 mM NaHCO3, 6 mM MgCl2¬, 5 mM TES, 5 mM Trehalose, 50 mM sucrose, and was pH’d to 7.2 with NaOH. The compound nerve of abdominal segments A3 and A4 was stimulated with suction electrodes while muscle 6 was impaled with 3 M KCl sharp microelectrodes (35–45 MΩ resistance). Stimuli were delivered using PClamp 9.0 software (Axon Instruments, Union City, CA) and all signals were collected at 10 KHz with a Dagan 8500 two-electrode voltage clamp amplifier (Minneapolis, MN) and filtered using a Gaussian filter with 3 KHz cutoff. Recordings were performed at room temperature, approximately 22 °C.

### Statistics

All comparisons were done using Welch’s t-test for unequal variances.

## Results

### Presynaptic Ena is required for Bouton and branch morphogenesis

Ena protein accumulates at the third instar NMJ [28] where it co-localizes with post-synaptic markers such as Dlg and Cactus (*Drosophila* homolog of IκB inhibitor) within the SSR [14]. However, clusters of Ena protein are also observed within motor axon terminals (“boutons”), indicating that Ena may also have a presynaptic function [14]. To address this possibility, we used a dominant LOF approach comparing NMJ synapse morphology in larvae with both wild-type and reduced Ena function. This method for reducing Ena function is based on Gal4-dependent expression of a high-affinity peptide ligand (*UAS-mito-FP*_*4*_) that sequesters Ena/VASP proteins to the surface of mitochondria, away from the normal sites of recruitment and activity [41]. This conditional approach precisely mimics *Drosophila ena* nulls in multiple cell types and developmental stages, including in oocytes and cultured cell lines [34, 42, 43].

**Fig. 1:**
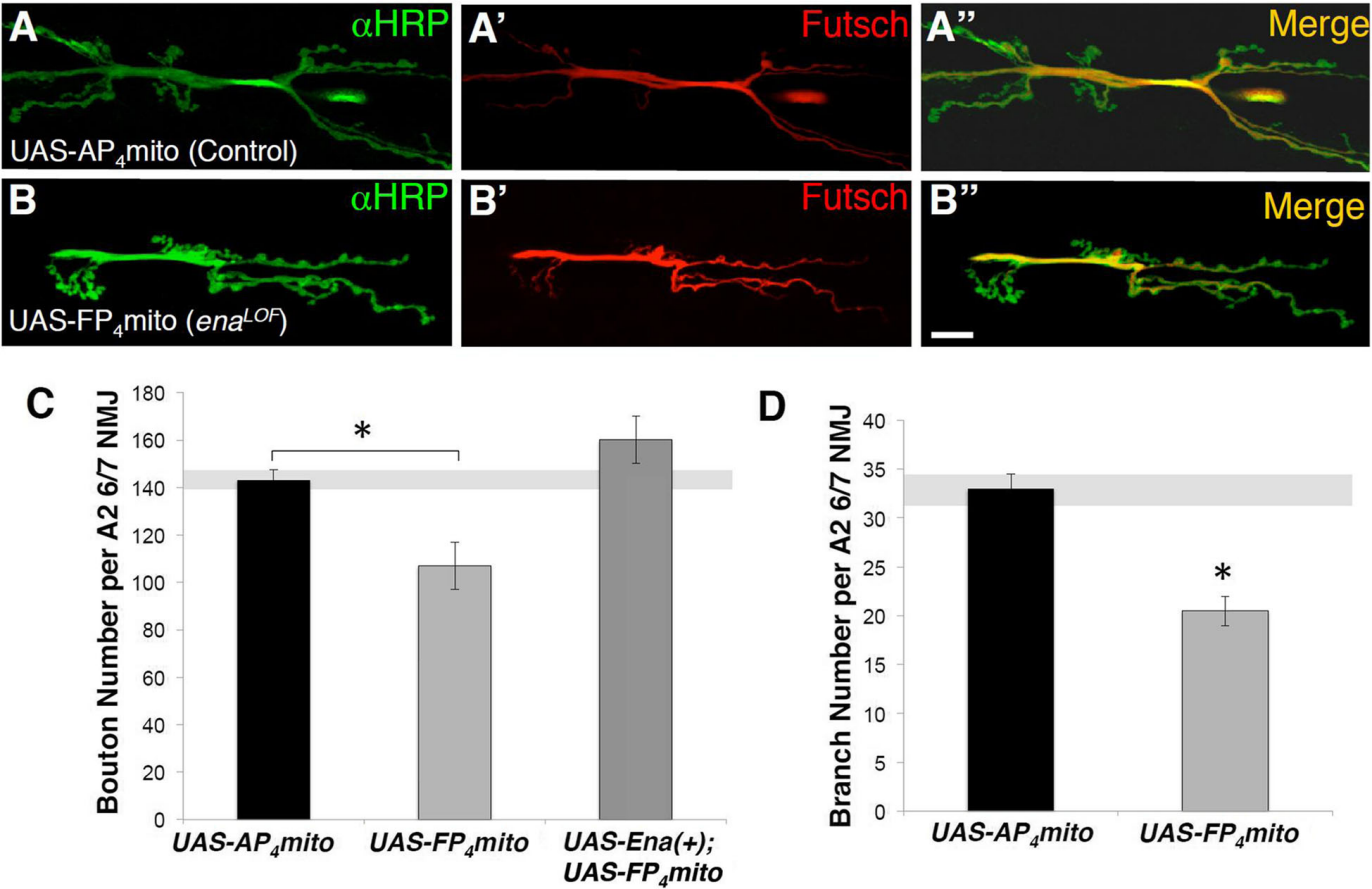
Presynaptic Ena expression is required to promote neuromuscular junction development. Fluorescence images (**a-b**) and quantification (**c-d**) of NMJs from muscle 6/7 in segment A2 of third-instar wandering larvae. Flies expressing *UAS-AP*_*4*_*mito* (control; **A-A"**) and *UAS-FP*_*4*_*mito* (*ena*^*LOF*^; **B-B"**) under the control of the neuronal *1407-GAL4* driver are shown stained with horseradish peroxidase (HRP; green, top panels), Futsch (red, middle panels), and with the HRP/Futsch channels merged (yellow, bottom panels). ***C*,** Quantification of synaptic 1b and 1 s bouton number in neuronal *ena*^*LOF*^ lines demonstrate a statistically significant decrease relative to control. Expression of *UAS-Ena(+)* under the control of *1407-GAL4* rescues the loss of bouton number in *ena*^*LOF*^ animals (**c**). ***D***, Branch number is also significantly decreased *ena*^*LOF*^. * *P* < 0.05, as determined by Welch’s t-test; error bars indicate ± s.e.m. of genotype; gray shading indicates ± s.e.m. of control; *n* ≥ 20 NMJs for all genotypes, scale = 20 μm

*ena*^*LOF*^ animals were generated by combining the *UAS-mito-FP*_*4*_ conditional transgene and the *1407-Gal4* driver, which drives expression in early neuroblasts stage 10/11 and subsequently most neurons of the CNS and all neurons of the peripheral nervous system [33]. Embryos were hatched at 18 °C and then shifted to 25 °C to delay *Gal4*-driven expression of *UAS-mito-FP*_*4*_ until first larval instar stage, thus avoiding early embryonic axon guidance phenotypes caused by *ena*^*LOF*^ (see Methods). We then examined synapses onto muscles 6 and 7 in mature third instar animals using antibodies against the neuron-specific HRP [44]. As a control, we expressed the *UAS-mito-AP*_*4*_ transgene, which bears a point mutation that eliminates Ena/VASP protein binding [34, 41]. We found that NMJ morphology was disrupted in *ena*^*LOF*^ animals (Fig. 1b) compared to controls (Fig. 1a). *ena*^*LOF*^ significantly reduced bouton and branch number (Fig. 1c and d), whereas these growth defects were prevented by co-expressing wild-type Ena (*UAS-Ena(+)*, Fig. 1c), confirming the specificity of the dominant LOF reagent and verifying an essential role for Ena in promoting NMJ expansion.

### Ena mediates the effects of Lar, Sdc, and Abl on NMJ growth

**Fig. 2:**
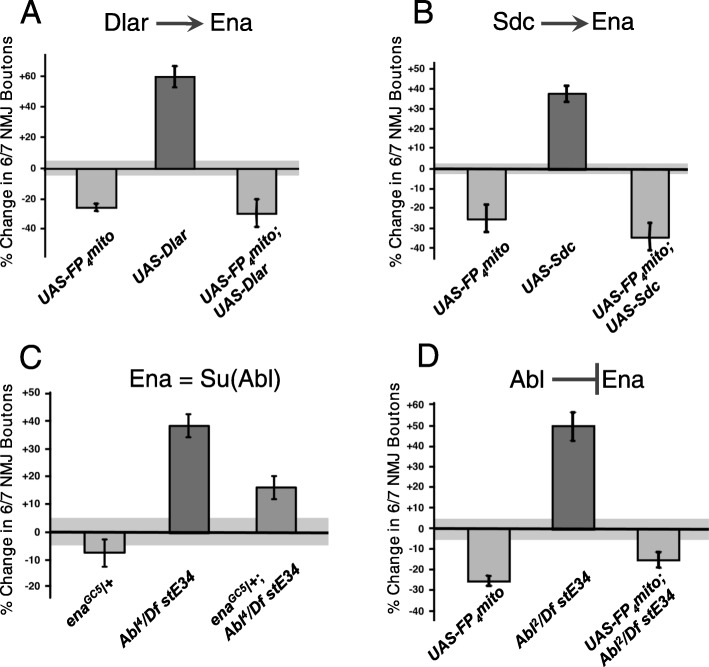
Ena is epistatic to Lar, Sdc, and Abl in NMJ growth. Gain of function (GOF) of the RPTP, Dlar (**a**), and associated HSPG ligand, Sdc (**b**), fail to rescue the bouton loss phenotype of *UAS-FP*_*4*_*mito* (*ena*^*LOF*^) flies when combined (DBL). Loss of the putative Ena suppressor Abl in *Abl*^*2*^*/Df stE34* increases bouton number (**c**), supporting the antagonistic interaction of Ena and Abl. Full suppression of the *Abl*^*2*^*/Df stE34* phenotype is observed in *UAS-FP*_*4*_*mito* (*ena*^*LOF*^) flies (**c**). Partial suppression of the *Abl*^*4*^*/Df stE34* phenotype is observed with haplosufficient *enaGC5/+* flies (**d**) indicating Ena is both downstream of and antagonized by Abl. Bouton number was determined by quantifying 1b and 1 s boutons. All results shown are statistically significant relative to control, with *P* < 0.05, as determined by Welch’s t-test. Error bars indicate ± s.e.m. of genotype; gray shading indicates ± s.e.m. of control; *n* ≥ 20 NMJs for all genotypes

The growth effects described above closely resemble those of *Dlar*^*LOF*^ mutations and are opposite of Abl mutations (Figure S1 A-H) [18, 32]. To examine the potential interactions between Ena and the Abl and Dlar pathways during NMJ development, we studied double mutants affecting Ena and Abl, Lar, or Syndecan (Sdc), a heparan sulfate proteoglycan (HSPG) that is a ligand of Dlar [29]. Overexpression (OE) of wild type transgenes for Dlar or Sdc with the *1407-Gal4* driver increased bouton numbers by 40–60%, opposite to the 20–30% decrease that is characteristic of the *ena*^*LOF*^ manipulation (Fig. 2a and b). When we combined *ena*^*LOF*^ with OE of Dlar or Sdc, we found the *ena* phenotype to be consistently epistatic (Fig. 2a and b), demonstrating that Ena is required for the function of components in the Dlar receptor pathway, consistent with our previous biochemical data showing that Ena phosphorylation is regulated by Dlar [25, 29].

Ena was first identified as a suppressor and substrate of the Abl kinase [19, 22, 23]. During NMJ development, Abl is required in neurons to restrict bouton and branch addition (Figure S1) [18]. Using the same double mutant strategy, we examined a strong *Abl*^*LOF*^ allele (*Abl*^*2*^*/Df(3)stE34*). As in the case of Dlar and Sdc, *ena*^*LOF*^ was epistatic to *Abl*^*LOF*^ (Fig. 2d), consistent with prior findings that Ena is regulated by Abl. A previous report failed to observe strong genetic suppression of an Abl allele that expresses a truncated but catalytically active protein (*Abl*^*1*^) [19] by dose reduction in *ena* alone [18]. We examined the genetic interaction between *ena* and *Abl* by combining a heterozygous null allele for *ena* (*enaGC5/+*) with the null *Abl*^*LOF*^ (*Abl*^*4*^*/Df(3)stE34*) and revealed a striking dose-dependent suppression of the Abl NMJ phenotype upon haploinsufficiency of Ena (Fig. 2c), further supporting a model where Ena functions downstream of both Dlar and Abl.

### Abl is epistatic to Dlar and Sdc for NMJ growth

**Fig. 3:**
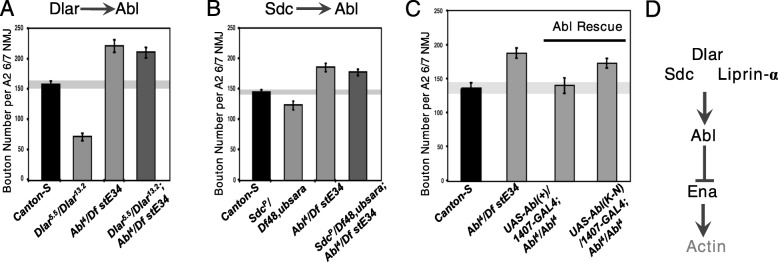
Synaptic Abl overgrowth phenotype is epistatic to Dlar and Sdc and requires the catalytic activity of Abl in the pre-synaptic compartment. Third-instar LOF mutants of Dlar (**a**) and Sdc (**b**), exhibit decreased bouton number in muscle 6/7 NMJs compared to Canton-S wild-type controls. The phenotypes of Lar and Sdc LOF mutants were suppressed by *Abl*^*LOF*^ (**a-b**), indicating that Abl is downstream of the Dlar pathway. Expression of *UAS-Abl(+)* under the control of the neuronal *1407-GAL4* driver rescued the bouton gain phenotype observed in Abl^*LOF*^ animals to levels observed in Canton-S flies (**c**). This indicates that pre-synaptic Abl is necessary and sufficient in synapse morphogenesis. Expression of kinase-dead Abl (*UAS-Abl(K-N)*) pre-synaptically failed to rescue *Abl*^*LOF*^ phenotypes (**c**), further supporting the requirement for Abl catalytic activity in synaptogenesis. ***D***, Schematic of the Dlar signaling pathway. Bouton number was determined by quantifying 1b and 1 s boutons. * *P* < 0.05, n.s. indicates not significant, as determined by Welch’s t-test; error bars indicate ± s.e.m. of genotype; gray shading indicates ± s.e.m. of control; n ≥ 20 NMJs for all genotypes

Although Abl and Dlar have reciprocal catalytic activity and both appear to require Ena, the epistatic relationship between Abl and the Dlar pathway during synapse morphogenesis has not been examined. To determine the genetic hierarchy of Abl activity with respect to Dlar pathway components, we again employed a classical double LOF approach. In each experimental case, the NMJ overgrowth phenotype of *Abl*^*4*^*/Df(3)stE34* was compared to strong alleles of Dlar (*Dlar*^*5.5*^*/Dlar*^*13.2*^) or Sdc (*Sdc*^*P*^*/Df(2)48,ub-Sara*), either individually or in double mutants. We observed that Abl was epistatic to both Sdc and Dlar (Fig. 3a and b; compare right-most double LOF bars to middle single LOF bars). Together with our other genetic data, this suggests that Ena functions as a key output for Abl downstream of the Dlar receptor complex during bouton addition. To indicate a role for Abl catalytic activity in this pathway, we performed rescue experiments where wild-type or kinase-dead (K-N) Abl transgenes were expressed under control of *1407-Gal4* in a *Abl*^*4*^*/Df(3)stE34* background. Although *UAS-Abl(+)* fully rescues the NMJ overgrowth induced by this strong *Abl*^*LOF*^ allele, *UAS-AblK-N* was unable to rescue the overgrowth (Fig. 3c). As Dlar catalytic activity is also required in this context [29], these observations are consistent with a model where Ena’s activity is regulated by a balance of Abl kinase and Dlar phosphatase activity, with potential effects on the actin cytoskeleton (Fig. 3d).

### Ena regulates active zone formation, spontaneous neurotransmission, and synaptic vesicle size and clustering

**Fig. 4:**
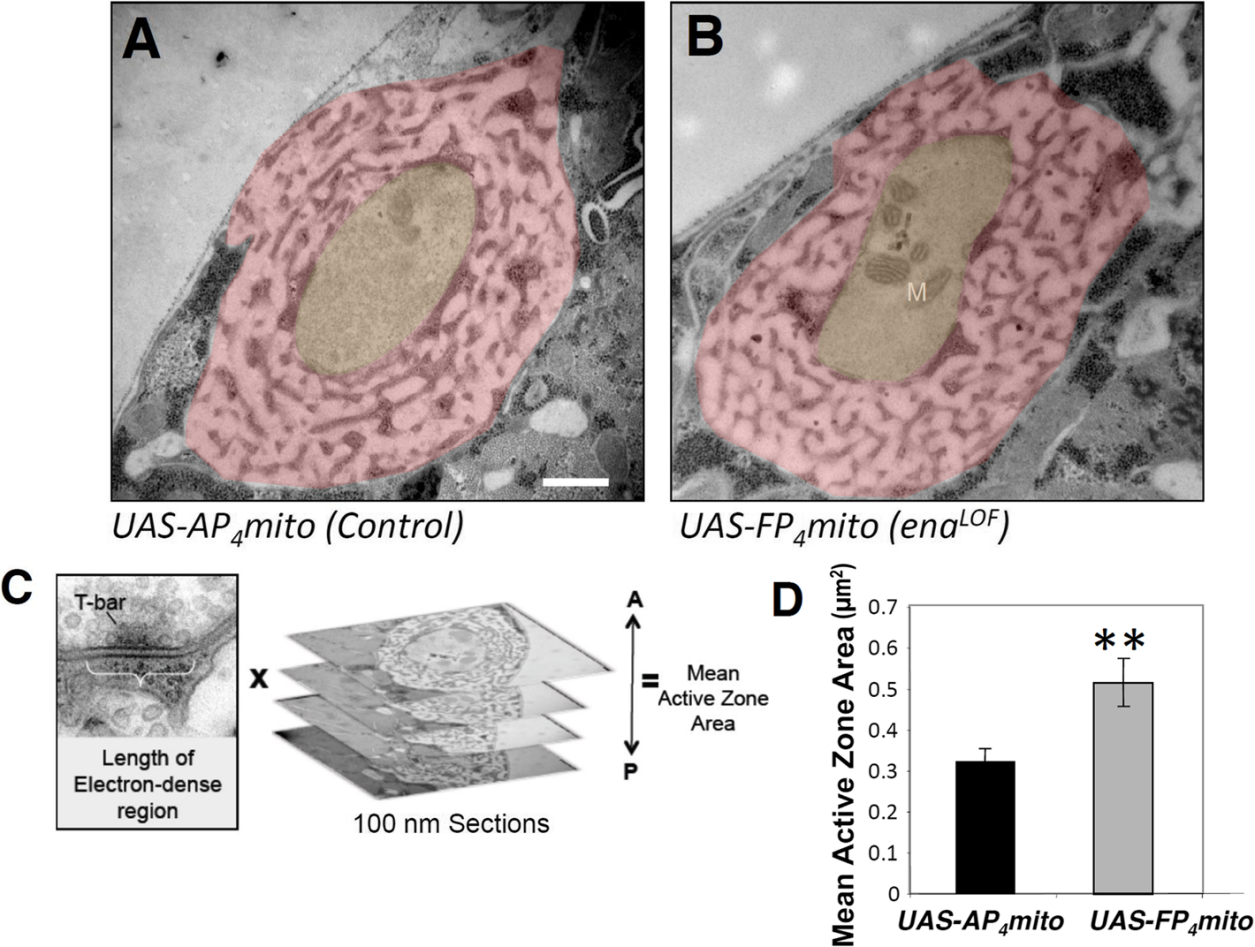
Presynaptic Ena regulates active zone structure. Electron micrographs of type 1b synaptic boutons at the 6/7 NMJ from flies expressing *UAS-AP*_*4*_*mito* (control, **a**) and *UAS-FP*_*4*_*mito* (*ena*^*LOF*^, **b**) under the control of the neuronal *1407-GAL4* driver were obtained to analyze gross, qualitative ultrastructure (**a,b**) and to quantify active zone area (**c,d**). Qualitative comparison revealed no catastrophic differences in SSR (pink shading) or bouton (yellow shading) morphology and/or size in *ena*^*LOF*^ (**b**) compared to controls (**a**). To determine quantitative phenotypes, mean active zone area was calculated by adding length of the electron dense region multiplied by the thickness of the serial sections (100 nm) for all sections spanning the active zone (**c**). ***D***, Mean active zone area is significantly increased in *ena*^*LOF*^. M indicates mitochondria; ** *P* < 0.01, as determined by Welch’s t-test; error bars indicate ± s.e.m. of genotype; gray shading indicates ± s.e.m. of control; *n* = 3 animals for all genotypes; scale bar = 500 nm

While cytoskeletal regulators like Abl and Ena are logical partners for Dlar during bouton morphogenesis, Dlar itself also controls the form and function of active zones at the NMJ [32]. To assess whether Ena acts as an output of Dlar in regulating active zone morphology, we examined synapse structure in *ena*^*LOF*^. We visualized NMJs by transmission electron microscopy in *1407-Gal4;UAS-mito-FP*_*4*_ compared to *1407-Gal4;UAS-mito-AP*_*4*_ and our initial qualitative analysis found no gross defects in *ena*^*LOF*^ in features such as the postsynaptic SSR or overall bouton morphology (Fig. 4a and b). More detailed quantitative analysis and measurement of the electron dense adhesive contact of the active zone in serial sections (indicated in Fig. 4c and d; see Methods) revealed a nearly two-fold increase in area in *ena*^*LOF*^ relative to control (Fig. 4 c and d). We furthermore performed structured-illumination analysis of the core active zone component Brp and found a comparable increase in Brp volume (Figure S2), consistent with our ultrastructural results. Interestingly, the increases in the size of the adhesive contact of the active zone observed upon *ena*^*LOF*^ are reminiscent of the Dlar phenotype as quantified with ultrastructure [32].

**Fig. 5:**
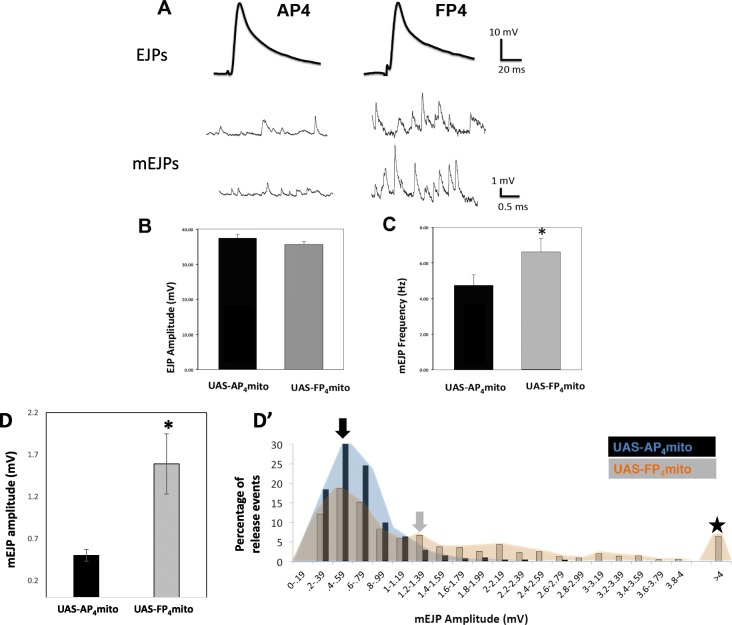
Presynaptic Ena function regulates spontaneous but not evoked glutamate release. ***a***, Current clamp recordings from muscle 6 (abdominal segments 3 and 4) revealed similar EJP amplitude and kinetics between the *AP*_*4*_*mito* control and *FP*_*4*_*mito* under the control of the neuronal *1407-GAL4* driver (top), yet very distinct spontaneous mEJPs (bottom). The mean EJP amplitude was not altered by *ena*^*LOF*^ (**b**), whereas mEJP frequency (**c**) and amplitude (**d**) were both significantly increased. ***D’***, An distribution of mEJP amplitude (an alternate depiction of data in **d**) shows a shift to the right in *ena*^*LOF*^ animals with abnormally high mEJPs (indicated by five-pointed star), and the mean mEJP amplitude was significantly increased (indicated by filled arrows). Results corresponding to control are depicted with a black arrow and blue distribution; results corresponding to *ena*^*LOF*^ are depicted with a gray arrow and orange distribution). * *P* < 0.05, as determined by Welch’s t-test, *n* = 3 animals and 6 NMJs for control, *n* = 4 animals and 7 NMJs for *ena*^*LOF*^

The active zone is the site at which neurotransmitter release occurs, and numerous studies have established the importance of proper active zone formation and morphology in synaptic function and efficacy [45, 46]. Thus, the defects in active zone size (Fig. 4**,**S2) in *ena*^*LOF*^ predicted an effect on neurotransmission. We recorded synaptic potentials from muscle fiber 6 in mature third instar larvae (see Methods) and observed either evoked excitatory junctional potentials (EJPs) or spontaneous “Miniature” EJPs (mEJPs). We found that *ena*^*LOF*^ did not affect the kinetics or amplitude of EJPs Fig. 5a and b) when compared to the *1407-Gal4; UAS-mito-AP*_*4*_ control or to the *1407-Gal4* or *UAS* parental strains. mEJPs, recorded in the absence of stimulation, were increased in both frequency and amplitude (Fig. 5c and d-d**’**), with some spontaneous release events greater than 4 mV (Fig. 5d**’,** five-pointed star). These results indicate a significant change in presynaptic function at the synapse. Quantal content (as defined by the ratio of the average EJP amplitude/average mEJP) at *ena*^*LOF*^ NMJs is less than half that of controls (Fig. 5d). Remarkably, the postsynaptic amplitudes of evoked EJPs have remained relatively unchanged, indicating the presence of a homeostatic mechanism at the NMJ that balances quantal release probability with quantal size.

**Fig. 6:**
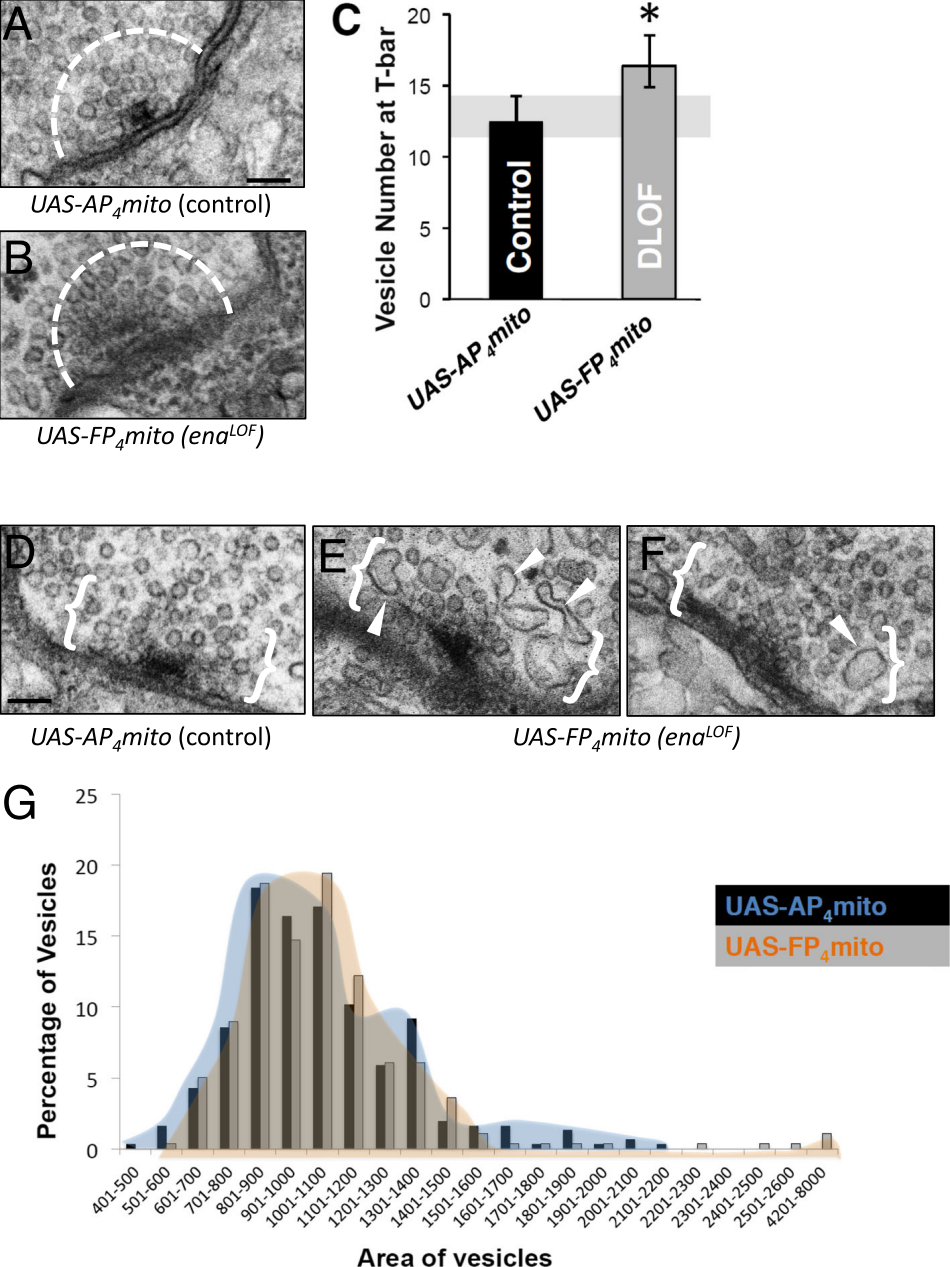
Ena is required to regulate clustering of synaptic vesicles, but not average vesicle size at the T-bar. ***a-g***, Analysis of electron micrographs of type 1b synaptic boutons at the 6/7 NMJ. Representative image of T-bar *AP*_*4*_*mito* control (**a**) and *FP*_*4*_*mito* (*ena*^*LOF*^, **b**) under control of the neuronal *1407-GAL4* driver. The dashed line (**a,b**) indicates 200 nm radius from the center of the T-bar. A significant increase in average SV number is observed in *FP*_*4*_*mito* animals within this region (*ena*^*LOF*^, **c**). ***d-f***, Abnormally-shaped and enlarged SVs within 200 nm from the electron dense adhesive contact of the active zone (indicated by white brackets) were observed in *FP*_*4*_*mito* (*ena*^*LOF*^, white arrow heads, **e-f**) in contrast to controls (**d**). Although average area of synaptic vesicles is unchanged in FP4mito (*ena*^*LOF*^) animals, the distribution of SV area (**g**) indicates rare large vesicles in these animals (orange distribution), which are not observed in control (blue distribution). * *P* < 0.05, as determined by Welch’s t-test; error bars indicate ± s.e.m. of genotype; gray shading indicates ± s.e.m. of control; *n* = 3 animals for all genotypes; scale = 100 nm

To better define ultrastructural features that might correlate with the increased mEJP frequency and extremely high amplitude miniature EJPs observed upon *ena*^*LOF*^ (Fig. 5 c and d), we examined vesicle size and distribution at the release sites. We quantified vesicle density and area within a 200 nm radius of neurotransmitter release sites (Fig. 6a-c) using methods previously reported [47]. We found the abundance of vesicles localized at active zones was significantly increased (Fig. 6c). Additionally, we found the average synaptic vesicle (SV) area with *ena*^*LOF*^ was comparable to that of control, but the size distribution was skewed with an extending tail of vesicles that were up to twice the size of the largest control vesicles (Fig. 6d-g). These phenotypes may account for the increased release probability as well as the increased amplitude observed in electrophysiological recordings.

## Discussion

The effector proteins and signaling pathways that regulate synaptogenesis via cytoskeletal assembly represent an important frontier [5, 6]. Our analysis of *Drosophila* Ena function at the larval NMJ tested the hypothesis that this conserved actin-regulatory factor plays a presynaptic role in sculpting synapse form and function.ecause zygotic Ena is essential for embryonic development and for actin assembly in many cell types, our test of this hypothesis required conditional disruption of Ena activity in larval neurons. Using a well-established method [34, 41–43], we find that presynaptic Ena is required for the addition of boutons and branches in motor axon terminals. Furthermore, this function of Ena is required for the expansion of terminal arbors that results from loss of Abl kinase activity. Finally, our analysis of synapse morphology and function reveals that Ena restricts the size of active zones, reminiscent of Dlar pathway function described in previous studies. Interestingly, Ena also restricts the number of SVs recruited to active zones, consistent with an increased frequency of spontaneous glutamate release when Ena is disrupted. These findings support a model where Ena acts downstream of Dlar and Abl during NMJ growth, but illustrates that Ena likely has additional Lar-independent functions during neurotransmission or SV trafficking.

Considering the logic of Ena function during synaptogenesis, our data reveal that Ena plays opposing roles in the pre- and post-synaptic compartments during motor terminal morphogenesis. On the postsynaptic side of the NMJ, Ena restricts motor neuron terminal morphogenesis through conserved actin-assembly domains that limit the expansion of the SSR [14]. In contrast, we find that on the presynaptic side, Ena functions to promote bouton and branch addition, along with its previously defined role in limiting ectopic satellite boutons [16]. Together, these findings indicate that presynaptic Ena is necessary both for addition of normal boutons and for blocking the formation of abnormal, undersized structures. In muscle, Ena levels and function are controlled by the microRNA miR-8; however, miR-8 does not regulate presynaptic Ena [14, 15]. Instead, presynaptic Ena appears to be controlled by signaling pathways, including Strip-Hippo [16] and Dlar. Dlar and miR-8 display mutually exclusive selectivity to pre- and postsynaptic compartments, respectively [14, 15, 29], raising the question of how the pre- and post-synaptic components of NMJ morphogenesis might be coordinated. Interestingly, a recent study has shown that Dlar is essential for NMJ morphogenesis and plasticity downstream of the retrograde Bone-Morphogenetic Protein (BMP) signaling pathway [40]. Thus, it is possible that Ena function downstream of Dlar is ultimately dependent on trans-synaptic communication. So far, the only known link between BMP and Dlar pathway function is the guanine nucleotide exchange factor (GEF) Trio that is downstream of the SMAD-family transcription factor Mothers Against Dpp (Mad) [40, 48]. Whether Trio acts upstream of Ena during NMJ morphogenesis is unknown. However, Trio-family GEFs are functionally coupled to Lar-family receptors in multiple contexts [49–51]. Moreover, Trio and Dlar appear to rely on the formin Diaphanous (Dia) during NMJ development [51], suggesting that bouton addition downstream of Dlar involves polymerization of bundled F-actin; if true, this would further suggest a protrusive context for Ena function in motor axon terminals that may involve filopodial extension to initiate bouton growth.

The Ena/VASP protein family plays multiple well-conserved roles during neural development, from the initiation of neuritogenesis to axon guidance and dendritic development [7–9]. In the context of growth cone navigation, Ena/VASP proteins are associated with several receptor families, including Lar-family receptors [25], Roundabouts (Robos) [24], and the UNC-40/DCC family of Netrin receptors [52]. Downstream of Netrin, Ena/VASP proteins regulate the actin-dependent protrusion of the leading edge membrane under control of protein kinase A (PKA) [53]. It has been previously suggested that Ena/VASP function in response to Netrin plays an important role in sculpting axon terminal branching patterns [54]. Although multiple axon guidance receptors are also known to regulate the process of synaptogenesis, growth cones can be quite distinct from synaptic terminals in both organization and dynamics. Indeed, the *Drosophila* NMJ expands during larval development by a process of terminal and interstitial budding of new presynaptic varicosities [55], and it is not clear if filopodial structures are required. This raises the fascinating question for future studies of whether Ena and upstream factors like Dlar and Abl play analogous roles in migrating growth cones and nascent boutons, or whether key signaling cassettes are redeployed with a fundamentally different outcome, perhaps due to distinct combinations of additional effector molecules.

In addition to identifying a presynaptic role for Ena/VASP proteins, our analysis also uncovers a contrast between the functions of Dlar and Ena at the active zone. Dlar and Liprin- α have been shown to regulate the morphology and function of active zones in *Drosophila* [32], consistent with the roles of their respective *Caenorhabditis elegans* homologs SYD-2 and PTP-3 [56–58]. Indeed, we find abnormal shape and increased size of active zones in *ena*^*LOF*^ NMJs that is highly reminiscent of both Dlar and Liprin-α mutants [32], consistent with a model where Ena acts downstream of Dlar to limit some aspect of active zone assembly or maintenance. However, loss of Dlar or Liprin-α reduces EJP amplitude, suggesting that this receptor and associated scaffolding molecule are required for the recruitment and/or function of other key active zone components that mediate Ca^2+^-dependent release of glutamate [32, 57]. In contrast, *ena*^*LOF*^ NMJs do not display decreased EJP amplitude or kinetics, but rather display higher rates of spontaneous glutamate release, i.e. increased mEJPs frequency. We also observed elevated mEJP amplitude, including rare cases where mEJP amplitude was very highly elevated, in *ena*^*LOF*^ NMJs. This suggests that Ena normally restricts active zone access or docking of SVs whose contents would be released spontaneously while facilitating the release of glutamate in a Ca^2+^-dependent manner (quantal content was reduced in the *ena*^*LOF*^).

The role of Ena as an actin regulator, combined with our evidence that Ena regulates active zone morphology, neurotransmitter release, and SV trafficking, is consistent with the well-established a role for actin as an active scaffold and SV organizer [4, 59, 60]. For instance, the *Drosophila* adaptor protein Nervous Wreck (Nwk), which directly binds the Arp 2/3-interactor WASP, is required for normal active zone density and synaptic transmission as well as SV clustering and endocytosis [61, 62], while the Arp 2/3-WAVE complex-interactor Cyfip [47], similarly regulates SVs at release sites in flies. We speculate that Nwk and Cyfip promote the formation of branched-actin filaments through their interactions with the Arp 2/3 complex. Given that Ena is known to inactivate Arp 2/3 and promote the formation of linear actin [8], it is possible that Ena counterbalances the effects of proteins such as Nwk and Cyfip. In general, actin is thought to regulate SV localization by tethering SVs [63–65]. Thus, the SV clustering defect we observe upon *ena*^*LOF*^ might indicate that Ena regulates this role of actin in SV organization. Furthermore, the the presynaptic actin cytoskeleton is known to be necessary for proper SV endocytosis [4, 59, 60]; we therefore speculate that the *ena*^*LOF*^ SV size phenotype likely reflects disruptions in endocytosis. In support of this, we note that the *ena*^*LOF*^ SV size defects resembles presynaptic phenotypes observed for mutants of *Dap160/Intersectin*, a known regulator of endocytosis that is also thought to be involved in actin regulation [66, 67].

It should be noted that Ena has been proposed as a negative regulator of Kinesin heavy chain and microtubule-dependent SV delivery at the NMJ [28], providing one possible mechanism to account for increased SV density. In addition, restriction of SV access to docking sites has been proposed for F-actin structures surrounding active zones in other species, such as lamprey [64, 68, 69]. Of course, it is also formally possible that the increased size of active zones in *ena*^*LOF*^ may simply result in higher numbers of docking sites per active zone. Either way, this means that while Ena may account for some of the Dlar pathway output, Ena cannot account for all Dlar pathway functions in this context. Recent analysis of different alleles of SYD-2/Liprin-α reveals that it is required for docking of SVs and maintaining normal levels of spontaneous release [57]. Although recordings of Dlar and Liprin-α mutants in *Drosophila* failed to detect altered mEJP properties [32], Abl mutants clearly alter mEJP frequency [18]. Interestingly, the impact of *Abl*^*LOF*^ on SV recruitment at AZs is opposite to *ena*^*LOF*^ [18]. Further physiology and SV trafficking studies will help elucidate this function.

## Conclusions

Here, we reveal multiple new presynaptic functions for *Drosophila* Ena at the larval NMJ, which was previously found to have roles in postsynaptic muscle cells. Presynaptic Ena promotes morphogenetic expansion of the larval NMJ through addition of synaptic boutons and branching in the motor axon terminal, in contrast to the role of postsynaptic Ena in restricting NMJ growth. Presynaptic Ena is epistatic to the RPTP Dlar and the associated HSPG Sdc and is furthermore epistatic to Abl, a non-receptor tyrosine kinase downstream of Dlar. Like Dlar, Ena regulates the formation of synaptic adhesion sites where active zone assembly occurs. However, electrophysiological and ultrastructural analysis of *ena*^*LOF*^ reveals additional roles for Ena in regulating recruitment, size distribution, and release of SVs at glutamatergic active zones which appear to be distinct from Dlar. We therefore show that both pre- and postsynaptic Ena have key effects on synaptic morphogenesis at the NMJ, but that the specific functions of Ena and its regulatory mechanisms are notably distinct between the two compartments.

## Supplementary information

**Additional file 1 **Supplemental **Figure 1.** Fluorescence images (A-F) and quantification (G-H) of NMJs from muscle 6/7 in segment A2 of third-instar wandering larvae. Wild-type flies (control; A-A") and *Abl* mutants (*Abl*^*2*^*/Df Ste34*; D-D") are shown stained with horseradish peroxidase (HRP; green, left panels), Futsch (red, middle panels), and with the HRP/Futsch channels merged (yellow, right panels). Staining with active zone markers Brp and endophilin (Endo) as well as the postsynaptic marker glutamate receptor subunit III (GluRIII) was qualitatively normal (B,C,E,F). *G,* Quantification of synaptic 1b and 1 s bouton number at muscle 4 (orange bars) and muscle 6/7 (blue bars). Abl mutant lines demonstrate an increase in bouton number relative to wild-type control (G). *H*, Branch number is also increased in *abl* mutants (E). Error bars indicate ± s.e.m. of genotype; orange and blue shading indicate ± s.e.m. of muscle 4 and 6/7 controls, respectively; *n* ≥ 20 NMJs for all genotypes, scale = 200 μm.

**Additional file 2 **Supplemental **Figure 2.** Structured illumination microscopy images (A-B) and quantification (C-D) of NMJs from muscle 6/7 in segment A2 of third-instar wandering larvae. Wild-type flies (control; A-A’) and *ena*^*LOF*^ (B-B′) are shown stained with HRP (red) and Brp (green) staining. *C, D,* Reconstruction and quantification of images showed that the density of Brp puncta (puncta per bouton area) was unchanged compared to control (C). However, Brp puncta volume was very significantly increased in *ena*^*LOF*^ compared to controls. ** *P* < 0.01, as determined by Welch’s test; error bars indicate ± s.e.m. of genotype; orange and blue shading indicate ± s.e.m. of muscle 4 and 6/7 controls, respectively; n ≥ 20 NMJs for all genotypes, scale = 1 μm.

## Data Availability

The datasets used and/or analyzed during the current study are included in the published manuscript and/or available from the following online sources: Dissertation authored by C.T.: http://id.lib.harvard.edu/alma/990121243320203941/catalog Harvard Dataverse repository: https://dataverse.harvard.edu/dataverse/presynaptic_ena

## Abbreviations

Abl: Abelson
BMP: Bone-Morphogenetic Protein
Dia: Diaphanous
Dlar: Leukocyte common antigen related
Dlg: Discs large
EJP: Excitatory junctional potentials
Ena: Enabled
EVH: Ena/VASP Homology
GEF: Guanine nucleotide exchange factor
GOF: Gain of function
HSPG: Heparan sulfate proteoglycan
LOF: Loss of function
Mad: Mothers Against Dpp
mEJP: Miniature excitatory junctional potentials
Mena: Mammalian Ena
NMJ: Neuromuscular junction
OE: Overexpression
PKA: Protein kinase A
Robos: Roundabouts
RPTP: Receptor protein tyrosine phosphatase
Sdc: Syndecan
SSR: Subsynaptic reticulum
SV: Synaptic vesicle
VASP: Vasodilator-Stimulated Protein
