# Supplementary figures and images for: Drosophila enabled promotes synapse morphogenesis and regulates active zone form and function
Source: Neural Dev. 2020 Mar 17;15:4. doi: 10.1186/s13064-020-00141-x (PMC7076993; doi:10.1186/s13064-020-00141-x)

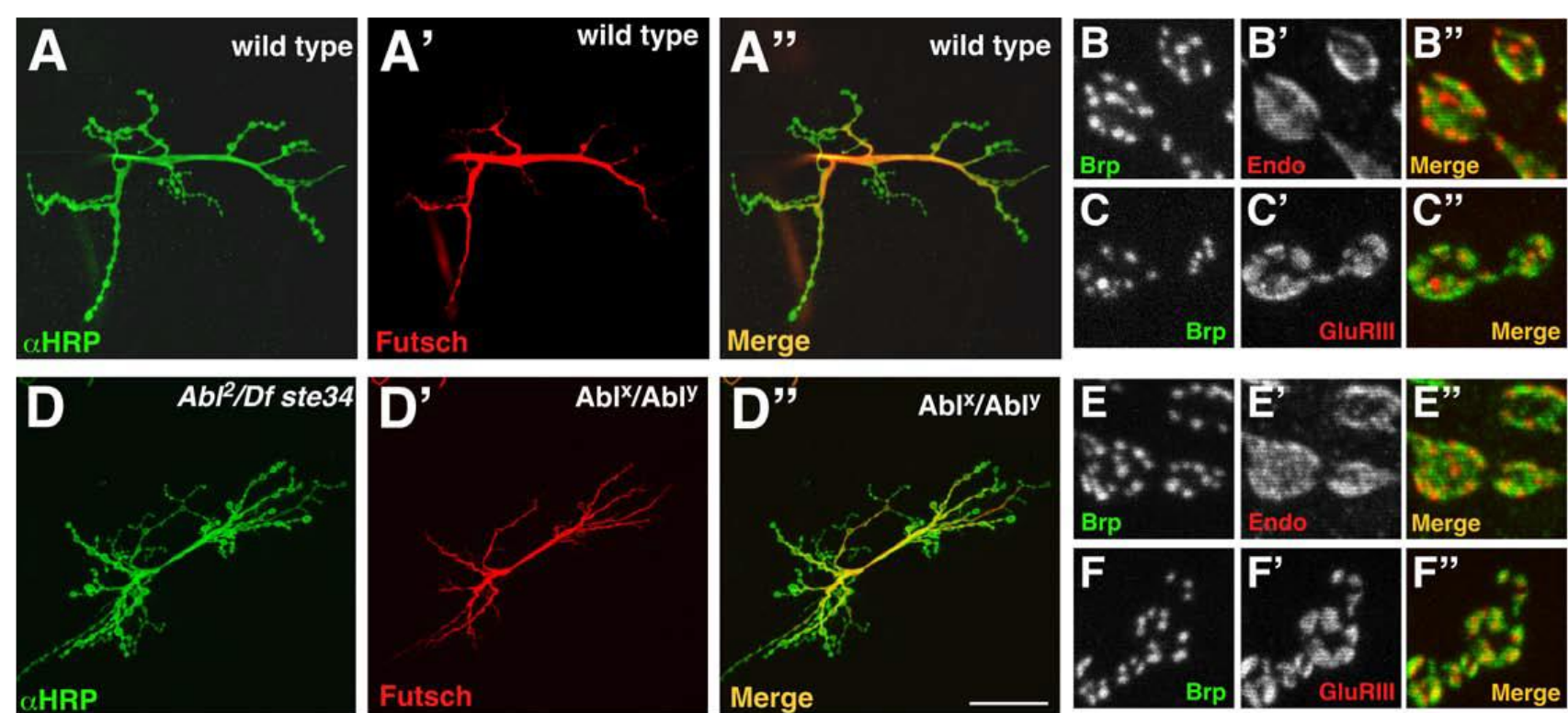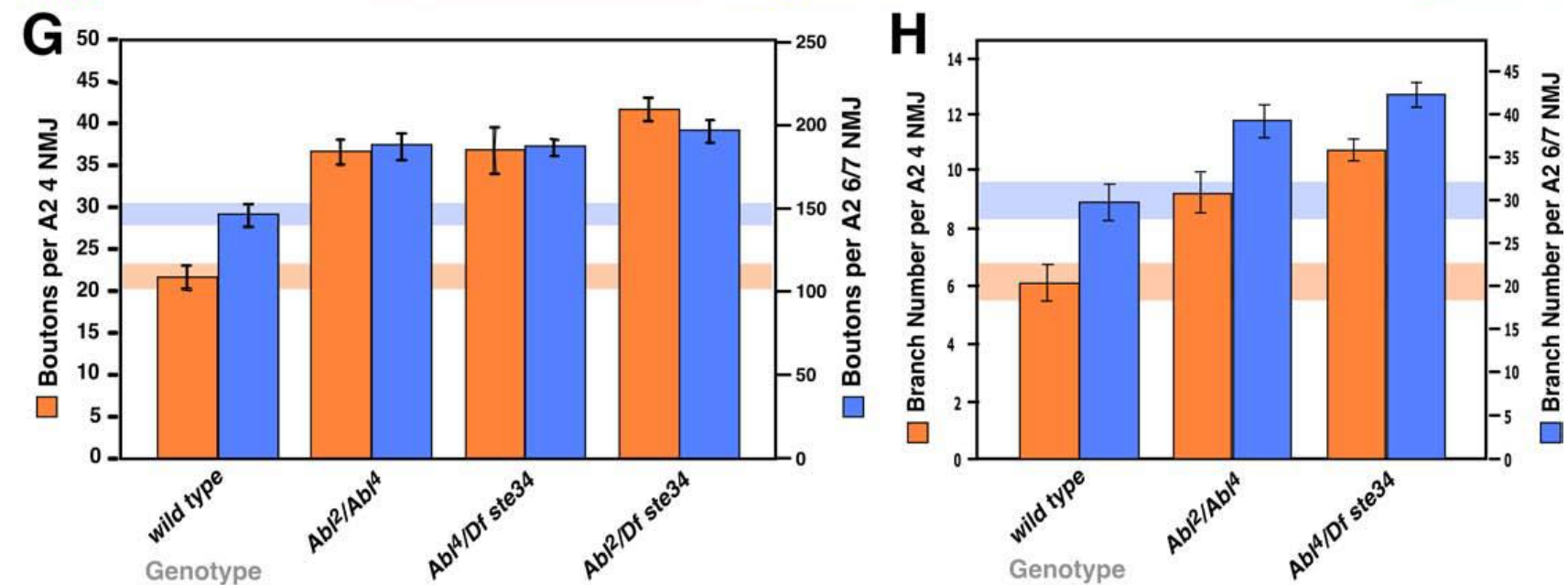

Supplement: Supplementary file 1 — Additional file 1 Supplemental Figure 1. Fluorescence images (A-F) and quantification (G-H) of NMJs from muscle 6/7 in segment A2 of third-instar wandering larvae. Wild-type flies (control; A-A") and Abl mutants (Abl2/Df Ste34; D-D") are shown stained with horseradish peroxidase (HRP; green, left panels), Futsch (red, middle panels), and with the HRP/Futsch channels merged (yellow, right panels). Staining with active zone markers Brp and endophilin (Endo) as well as the postsynaptic marker glutamate receptor subunit III (GluRIII) was qualitatively normal (B,C,E,F). G, Quantification of synaptic 1b and 1 s bouton number at muscle 4 (orange bars) and muscle 6/7 (blue bars). Abl mutant lines demonstrate an increase in bouton number relative to wild-type control (G). H, Branch number is also increased in abl mutants (E). Error bars indicate ± s.e.m. of genotype; orange and blue shading indicate ± s.e.m. of muscle 4 and 6/7 controls, respectively; n ≥ 20 NMJs for all genotypes, scale = 200 μm. [file 13064_2020_141_MOESM1_ESM.pdf]

AP<sub>4</sub>mito (control)

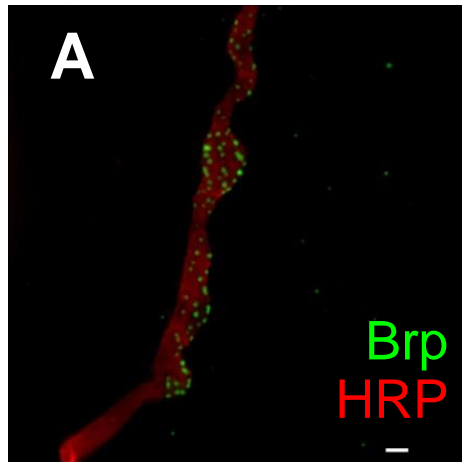

FP<sub>4</sub>mito (*ena*<sup>LOF</sup>)

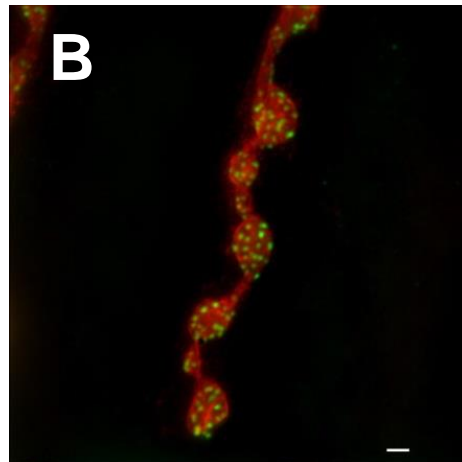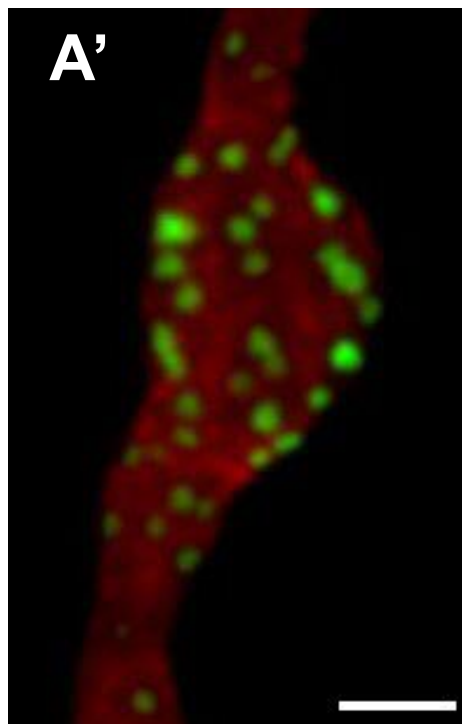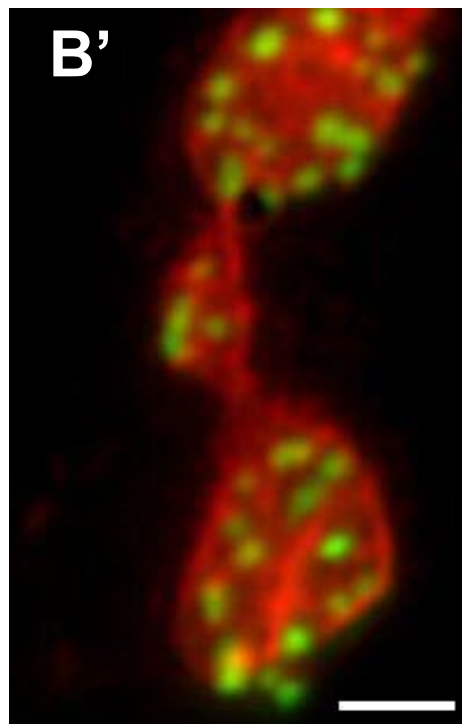

**C**

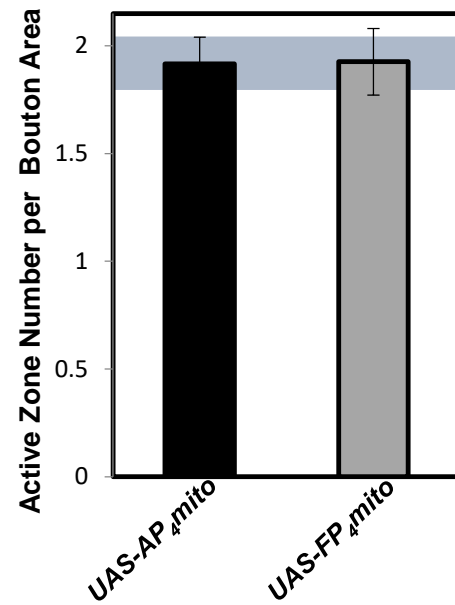

**D**

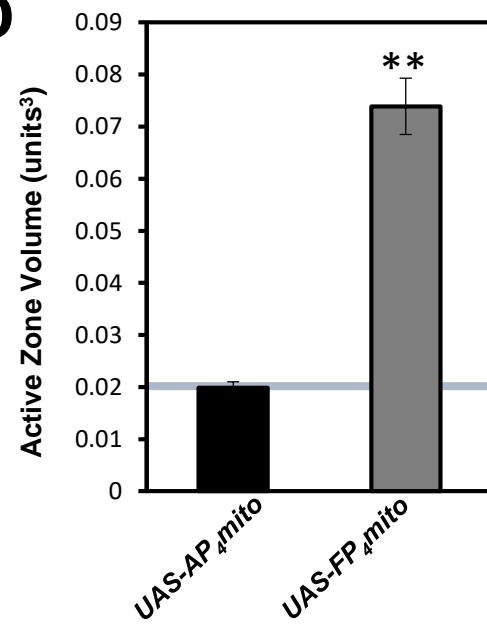

Supplement: Supplementary file 2 — Additional file 2 Supplemental Figure 2. Structured illumination microscopy images (A-B) and quantification (C-D) of NMJs from muscle 6/7 in segment A2 of third-instar wandering larvae. Wild-type flies (control; A-A’) and enaLOF (B-B′) are shown stained with HRP (red) and Brp (green) staining. C, D, Reconstruction and quantification of images showed that the density of Brp puncta (puncta per bouton area) was unchanged compared to control (C). However, Brp puncta volume was very significantly increased in enaLOF compared to controls. ** P < 0.01, as determined by Welch’s test; error bars indicate ± s.e.m. of genotype; orange and blue shading indicate ± s.e.m. of muscle 4 and 6/7 controls, respectively; n ≥ 20 NMJs for all genotypes, scale = 1 μm. [file 13064_2020_141_MOESM2_ESM.pdf]
